# Effect of Alirocumab on Coronary Calcification in Patients With Coronary Artery Disease

**DOI:** 10.3389/fcvm.2022.907662

**Published:** 2022-05-06

**Authors:** Fei Gao, Yue Ping Li, Xiao Teng Ma, Zhi Jian Wang, Dong Mei Shi, Yu Jie Zhou

**Affiliations:** Department of Cardiology, Beijing Anzhen Hospital, Capital Medical University, Beijing, China

**Keywords:** coronary artery disease, coronary calcification, PCSK9 inhibition therapy, alirocumab, statin

## Abstract

**Background:**

Proprotein convertase subtilisin kexin type 9 (PCSK9) inhibitors have been documented with significantly reduction in LDL cholesterol levels and cardiovascular events. However, evidence regarding the impact of PCSK9 inhibitors on coronary calcification is limited.

**Methods:**

Eligible patients with intermediate coronary lesions and elevated LDL cholesterol values were randomized to either alirocumab 75 mg Q2W plus statin (atorvastatin 20 mg/day or rosuvastatin 10 mg/day) therapy or standard statin therapy. Calcium score based on coronary computed tomographic angiography at baseline and follow up were compared.

**Results:**

Compared with baseline levels, LDL cholesterol were significantly decreased in both groups, while the absolute reduction of LDL cholesterol levels were higher in patients treated with alirocumab (1.69 ± 0.52 vs. 0.92 ± 0.60, *P* < 0.0001). Additionally, patients in alirocumab group demonstrated a significant reduction of Lp(a) levels, whereas it was not observed in the standard statin group. Notably, greater increases in the percentage changes of CAC score (10.6% [6.3–23.3] vs. 2.9% [−6.7–8.3]; *P* < 0.0001) were observed in the statin group compared to the alirocumab group. Consistently, CAC progression was significantly lower in the alirocumab group than in the standard statin group (0.6 ± 2.2% vs. 2.7 ± 2.3%; *P* = 0.002).

**Conclusions:**

Study indicated that administration of the PCSK9 inhibitors to statins produced significantly lower rate of CAC progression in patients with coronary artery disease. Further studies with CAC progression and their clinical outcomes are needed.

**Trial Registration:**

ClinicalTrials.gov, Identifier: NCT04851769.

## Introduction

Despite rapid advances in the treatment of coronary artery disease (CAD), coronary artery calcification (CAC) remains a challenging clinical problem, and it is directly correlated with future cardiovascular events and confers an increased risk of procedural complications (1, 2). To date there are no pharmacotherapeutics that can be proved to prevent or inhibit CAC progression. Statins, while being the cornerstone for effective treatment of CAD, have failed to demonstrate a positive effect on CAC progression (3, 4).

Emerging evidence indicates that proprotein convertase subtilisin kexin type 9 (PCSK9) may contribute to the cardiovascular calcification process (5). Patients with the highest PCSK9 concentrations have the highest CAC score (6, 7) and a PCSK9 loss-of-function variant is protective for aortic valve calcification (8). Additionally, translational studies reveal that plasma lipoproteins, particularly Low-density lipoprotein (LDL) cholesterol and lipoprotein(a), are involved in the pathogenesis of CAC (7, 9). As a novel lipid lowering agent, PCSK9 inhibitors have been demonstrated with significantly reduction in LDL cholesterol, lipoprotein(a) [Lp(a)] and cardiovascular events (10, 11). This has led to an interesting notion that whether PCSK9 inhibitors have a potential role in inhibiting cardiovascular calcification.

Serial intravascular imaging studies demonstrated that PCSK9 inhibitors exerted favorable effects against the progression of coronary atherosclerosis (12, 13), whereas CAC progression was not evaluated in these studies. Computed tomography calcium scoring system enables whole-heart quantification of coronary calcification, and current consensus recommend the use of Agatston scoring as the preferred measurement of assessing CAC progression (14). Therefore, to investigate the effects of PCSK9 inhibitors on CAC progression, we performed a calcium score analysis of the randomized control study comparing PCSK9 inhibitors vs. standard statin therapy in patients with coronary artery disease.

## Methods

### Study Participants

The study is a secondary analysis of the randomized controlled study involving patients with intermediate coronary lesions (50–70% diameter stenosis) and elevated LDL cholesterol values despite taking standard statin therapy. The detailed study design was published before (15). In brief, a total of 61 patients were randomized to either the alirocumab arm (30 patients) or the standard care arm (31 patients). Eligible patients included those who were (I) diagnosed with acute coronary syndrome (ACS) or stable coronary artery disease, (II) identified with at least one intermediate lesion (50–70% diameter stenosis) on *de novo* coronary arteries, (III) unable to achieve the target LDL cholesterol levels (LDL cholesterol ≥ 1.81 mmol/L [≥70 mg/dL] for patients with ACS, or ≥2.59 mmol/L [≥100 mg/dL] for non-ACS patients) despite taking statins (rosuvastatin 10 mg/day or atorvastatin 20 mg/day) or with maximally tolerated statin therapy. Among the overall cohort, patients with coronary computed tomographic angiography (CTA) at baseline were involved in this analysis. Patients with prior usage of PCSK9 inhibitors or received balloon angioplasty or stent implantation were excluded. Patients in the alirocumab arm received alirocumab 75 mg every two weeks on top of standard statin therapy (atorvastatin 20 mg/day or rosuvastatin 10 mg/day) for at least 36 weeks. Patients in the standard care arm continued to receive atorvastatin 20 mg/day or rosuvastatin 10 mg/day. Statin dose escalation or the addition of other non-statin lipid-lowering therapies could be considered by their responsible physicians to achieve the target LDL cholesterol levels. All patients were monitored and evaluated for medical adherence to lipid lowering therapy during the study period. Follow-up coronary computed tomographic angiography was carried out at 15 ± 1 months after the initiation of treatment in both arms. LDL cholesterol, HDL cholesterol, and triglycerides were measured by the central laboratory. LDL cholesterol was calculated according to the Friedewald formula (16). Lp(a) was quantified using a particle-enhanced immunonephelometric method (Siemens Healthcare, Germany). All participants provided informed consent, and the study was approved by the local medical ethics committee.

### CAC Score Measurement

All coronary CTAs were performed in accordance with Society of Cardiovascular Computed Tomography guidelines (17). CAC was scored using the Agatston method (18), which was calculated for each calcified lesion. The total CAC score was calculated as the sum of all arteries including the left main artery (LM), the left circumflex artery (LCX), the left anterior descending artery (LAD), and the right coronary artery (RCA). The CAC score were evaluated by a single radiologist who was unaware of the purpose of the study.

### Study Outcomes Ascertainment

The primary outcome of this study was CAC progression over the study period. Secondary outcome was the percentage change between the baseline and follow-up CAC scores. CAC progression is the development of CAC during baseline and follow-up CT scans. To adjust for the skewed distribution, CAC progression was calculated as [log(CAC + 1) at the follow up]–[log(CAC + 1) at the baseline], and was then retransformed to depict the percentage change in CAC.

### Statistical Analysis

Continuous variables are reported as the means ± standard deviations or median (interquartile ranges), whereas categorical variables are presented as counts and percentages. Comparisons of categorical variables were performed by the Fisher's exact test. Continuous variables of the baseline and follow-up were compared with the Wilcoxon signed-rank test. Continuous variables between the statin group and the PCSK9 inhibitor group were compared with the Mann–Whitney *U* test. The total CAC burden before the study treatment was stratified into the following score categories: <400 (mild to moderate) and ≥400 (severe) (18). Linear regression analysis was used to evaluate the association of CAC progression with plasma lipoproteins. Age, sex as well as cardiovascular risk factors (hypertension, diabetes, smoking) were included in multivariate analysis. Due to the non-normal positively skewed distribution of the CAC score, we lg-transformed it for multiple linear regression [CAC → lg (CAC+1)]. A two-tailed test *P* value < 0.05 was considered as statistically significant. The SPSS Statistics 25.0 package was used.

## Results

A total of 51 eligible patients (28 patients in the standard arm and 23 patients in the alirocumab arm) with complete baseline and follow-up coronary CTA were analyzed. Over two thirds of patients (19/28) in the standard treatment arm had statin dose adjustments and nearly half of the patients (13/28) received ezetimibe and statin combination therapy. The baseline characteristics were listed in Table 1. Although the proportion of current smoker, female and patients with prior stroke in the alirocumab is numerically higher than the standard group, there was no statistically significant difference between the two groups (Table 1). Of note, the study subjects had a high prevalence of guideline recommended medical therapies in both groups. Particularly, antiplatelet therapy was prescribed 100% to the participants, and over 90% of them received beta blockers.

**Table 1:** Baseline characteristics.

	**Standard statin (*N* = 28)**	**Alirocumab** **(*N* = 23)**	***P* value**
Age, years	60.4 ± 9.5	62.9 ± 8.6	0.32
Male, % (N)	75.0 (21)	65.2 (15)	0.45
Diabetes, % (N)	25.0 (7)	21.7 (5)	0.78
Current smoker, % (N)	21.4 (6)	30.4 (7)	0.46
Hypertension, % (N)	64.3 (18)	60.9 (14)	0.80
Prior MI, % (N)	10.7 (3)	13.0 (3)	0.80
Prior stroke, % (N)	3.6 (1)	13.0 (3)	0.21
ACS, % (N)	42.9 (12)	34.8 (8)	0.56
Antiplatelet, % (N)	100 (28)	100 (23)	-
Beta-blocker, % (N)	91.3 (26)	92.9 (21)	0.84
ACEI/ARB, % (N)	67.9 (19)	56.5 (13)	0.41
Chronic statin before enrollment, % (N)	28.6 (8)	21.7 (5)	0.58

*ACS, acute coronary syndrome; MI, myocardial infarction; ACEI/ARB, angiotensin-converting enzyme inhibitors/angiotensin receptor blockers*.

### Changes in Biochemical Parameters

Biochemical parameters during the study period are summarized in Table 2. At baseline, no significant differences were observed between the standard care arm and the alirocumab arm. After treatment, LDL cholesterol levels were significantly decreased in both groups compared with baseline, whereas the absolute reduction of LDL cholesterol levels were significantly higher in patients treated with alirocumab (1.69 ± 0.52 vs. 0.92 ± 0.60, *P* < 0.0001). Additionally, patients in alirocumab group demonstrated a statistically significant reduction of Lp(a) and triglyceride levels. However, the reduction of Lp(a) and triglyceride levels was not observed in the standard care group. The value of C-reactive protein (CRP) decreased in both alirocumab and standard care groups, and the absolute changes were not statistically different between the two groups.

**Table 2:** Biochemical parameters.

	**Standard statin** **(*N* = 28)**	**Alirocumab** **(*N* = 23)**	***P* Value**
**LDL cholesterol, mmol/L**			
Baseline	3.10 ± 0.96	2.99 ± 0.78	0.81
At follow-up	2.19 ± 0.71^*^	1.28 ± 0.40^*^	<0.0001
Changes from baseline	−0.92 ± 0.60	−1.69 ± 0.52	<0.0001
**HDL cholesterol, mmol/L**			
Baseline	1.29 ± 0.42	1.42 ± 0.32	0.16
At follow-up	1.37 ± 0.35	1.51 ± 0.37	0.35
Changes from baseline	0.09 ± 0.16	0.09 ± 0.12	0.74
**Triglycerides, mmol/L**			
Baseline	1.50 (1.17 to 2.19)	1.63 (1.09 to 2.31)	0.70
At follow-up	1.48 (1.15 to 1.96)	1.40 (0.98 to 1.91)^*^	0.40
Changes from baseline	−0.03 (−0.30 to 0.28)	−0.24 (−0.81 to 0.20)	0.04
**CRP, mg/L**			
Baseline	1.71 (0.90 to 3.42)	1.62 (0.57 to 3.73)	0.68
At follow-up	1.40 (0.89 to 2.88)^*^	1.49 (0.72 to 2.91)^**^	0.66
Changes from baseline	0.30 (−0.66 to 1.43)	0.14 (−0.51 to 0.89)	0.08
**Lp(a), nmol/L**			
Baseline	28.2 (11.6 to 69.4)	23 (11.0 to 65.0)	0.53
At follow-up	26.0 (11.3 to 69.0)	20.3 (8.8 to 55.2)^*^	0.38
Changes from baseline	−0.6 (−2.3 to 0.6)	−3.1 (−18.0 to −1.9)	0.001

*CRP, C-reactive protein; LDL, low-density lipoprotein; HDL, high-density lipoprotein; Lp(a), lipoprotein*.

* *P < 0.0001 compared to the baseline levels*.

** *P < 0.01 compared to the baseline levels*.

### Changes in the CAC Score

**Figure 1:**
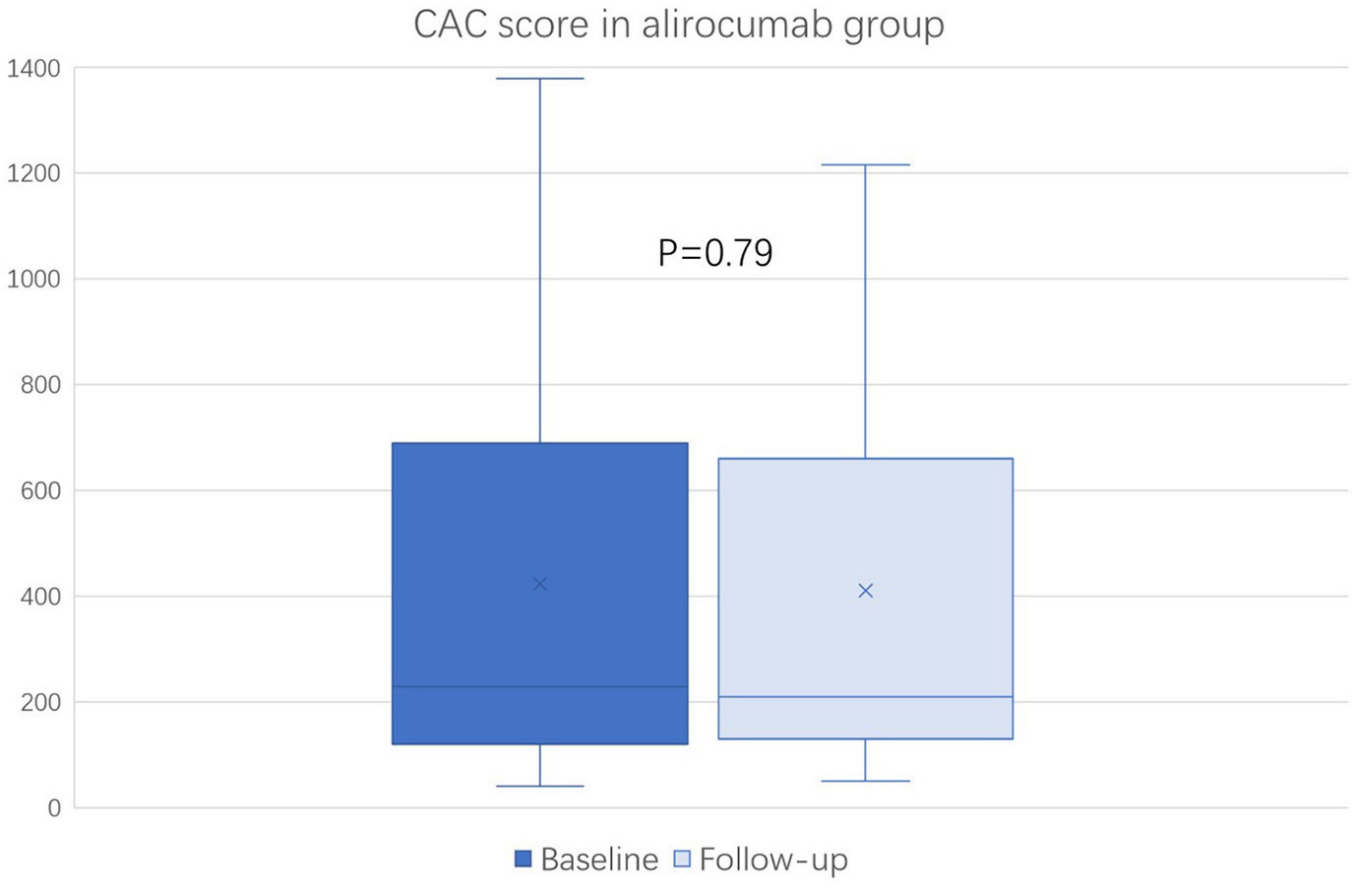
Comparison of coronary artery calcium (CAC) score between the baseline and follow-up in the alirocumab (*N* = 23). CAC scores between baseline and follow-up were compared with the Wilcoxon signed-rank test.

**Figure 2:**
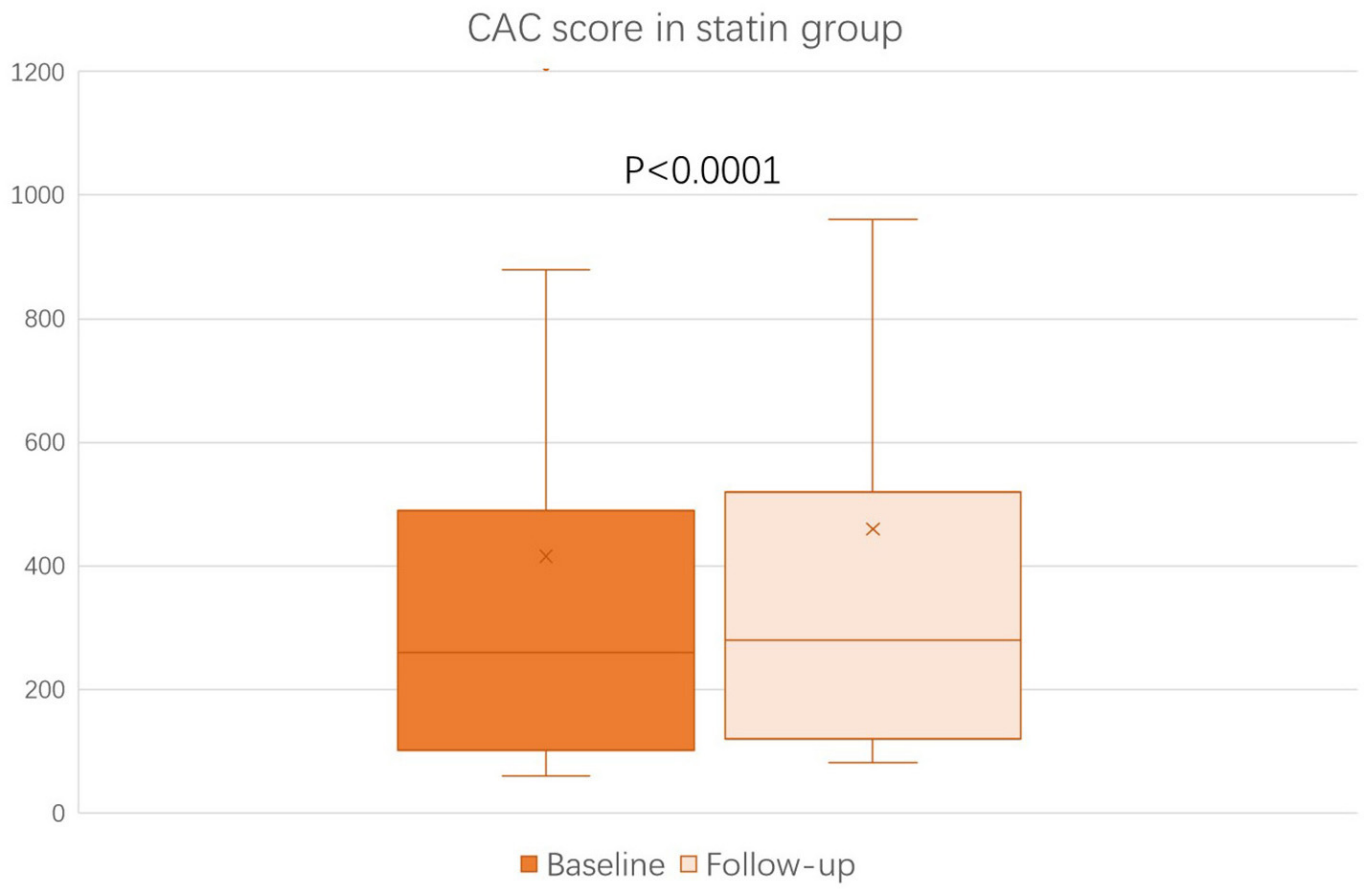
Comparison of coronary artery calcium (CAC) score between the baseline and follow-up in the statin (*N* = 28) group. CAC scores between baseline and follow-up were compared with the Wilcoxon signed-rank test.

**Figure 3:**
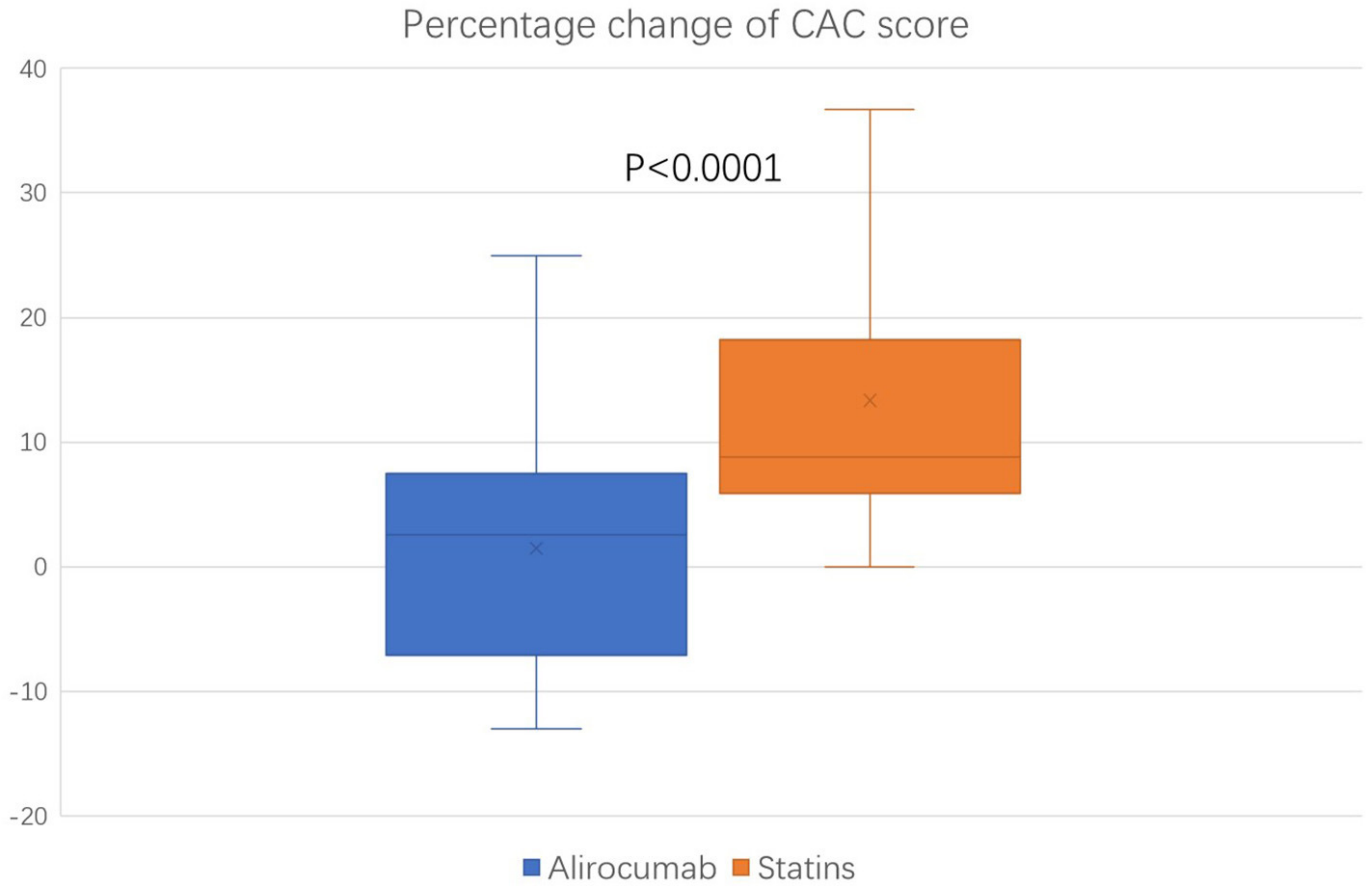
Comparison of percentage change in coronary artery calcium (CAC) score between the alirocumab (*N* = 23) and the statin (*N* = 28) group. Percentage changes of CAC score between the statin group and the PCSK9 inhibitor group were compared with the Mann–Whitney *U* test.

The baseline (before the initiation of study treatment) and the follow-up CAC score between the alirocumab and statin groups was shown in Figures 1, 2, respectively. In the statin group, the follow-up CAC score was significantly increased compared to the baseline values (267 [122–550] vs. 255 [100–512]; *P* < 0.0001), whereas in the alirocumab group the CAC score did not differ between the baseline and follow-up (210 [132–660] vs. 230 [120–690]; *P* = 0.79). Importantly, significantly greater increases in the percentage changes of CAC score (10.6% [6.3–23.3] vs. 2.9% [−6.7–8.3]; *P* < 0.0001) were observed in the statin group compared to the alirocumab group (Figure 3). Consistently, the log-transformed CAC progression was significantly lower in the alirocumab group than in the standard statin group (0.6 ± 2.2% vs. 2.7 ± 2.3%; *P* = 0.002).

### Impact of Baseline CAC Score on CAC Progression

To assess whether CAC progression was influenced by the severity of coronary calcification at baseline, the study subjects were then stratified by their baseline CAC (≥ 400 vs. <400). The association of alirocumab prescription and lower CAC progression was consistent for subjects with either baseline CAC scores <400 or ≥400, whereas for subjects with CAC score ≥400 the rate of CAC progression was considerably lower for both statin and alirocumab group (Table 3).

**Table 3:** Stratified comparison of CAC progression with baseline CAC score.

	**Standard statin (*N* = 28)**	**Alirocumab** **(*N* = 23)**	***P* Value**
**CAC progression (%)**			
All (*N* = 51)	2.7 ± 2.3	0.6 ± 2.2	0.002
CAC <400 (*N* = 31)	3.5 ± 2.7	1.2 ± 2.6	0.024
CAC > 400 (*N* = 20)	1.5 ± 0.9	−0.3 ± 1.4	0.001

*CAC, coronary artery calcium*.

### Relation of Plasma Lipoproteins and CAC Progression

To evaluate the association between the changes of plasma lipoproteins and the CAC progression, we performed an unadjusted linear analysis for Lp(a), triglyceride, LDL cholesterol, high-density lipoprotein cholesterol and CRP levels in the whole cohort. The study demonstrated that CAC progression was only significantly associated with the changes of Lp(a) levels (0.09 [0.03; 0.16], *p* = 0.007), whereas the association was not found with other parameters (Table 4). After multivariate adjustments with age, sex and cardiovascular risk factors, the association between the changes of Lp(a) and CAC progression remained significant (0.10 [0.03 0.16], *p* = 0.009).

**Table 4:** Association between the changes of plasma lipoproteins and CAC progression.

**R^**2**^ = 0.18**	**Beta [95% CI]**	***P* Value**
Lp(a)	0.09 [0.03, 0.16]	0.007
LDL cholesterol	0.74 [−0.36, 1.87]	0.18
HDL cholesterol	−0.99 [−6.11, 4.13]	0.69
Triglycerides	−0.38 [−1.67, 0.90]	0.55
CRP	0.41 [−0.61, 1.68]	0.23

*Lp(a), lipoprotein(a); CRP, C-reactive protein; LDL, low-density lipoprotein; HDL, high-density lipoprotein; Lp(a), lipoprotein*.

## Discussion

The results of our study indicated that the use of alirocumab was associated with a significantly lower progression of coronary artery calcification compared with standard lipid lowering therapy. The study also demonstrated a significant Lp(a) lowering effect with alirocumab, and the extent of CAC progression was correlated with the Lp(a) changes.

Coronary atherosclerosis plaque progression is associated with intimal calcification (19), and the calcifications become larger as plaques progress (20). Lipid lowering therapy with statins have been documented to improve plaques stability by decreasing plaque burden, but increasing plaque calcification (3, 4). Previous studies indicated that elevated PCSK9 levels correlated with increased risk of coronary artery calcification in patients with familial hypercholesterolemia under statin treatment (7, 21). Subgroup analysis of the FOURIER study revealed that the PCSK9 inhibitors could decrease the incidence of calcific aortic valve disease (5). To date, there is a gap of evidence regarding the impact of PCSK9 inhibition on coronary artery calcification. To the best of our knowledge, this was the first study to investigate the effects of PCSK9 inhibitors on CAC progression in a randomized controlled study comparing PCSK9 inhibitors vs. standard statins therapy in patients with coronary artery disease. Previous intravascular ultrasound studies demonstrated that the use of PCSK9 inhibitors resulted in a greater reduction in total atheroma volume compared to statin monotherapy (12, 13), and the primary results of our study with optical coherence tomography analysis showed that the addition of PCSK9 inhibitors to statins were associated with greater increases in fibrous cap thickness and reduced lipid arc (15, 22). The GLAGOV trial showed that the use of evolocumab vs. statins did not produce differential changes in the percentage of plaque occupied by dense calcium assessed by virtual histology ultrasound (12). However, intravascular ultrasound tends to overestimate calcification due to echo shadow and it can only identify the distribution of calcium present in a plaque (23). CAC measured by Agatston score enables the whole-heart quantification of coronary calcification, and it is considered as the preferred measurement of assessing CAC progression (14, 23). Ikegami et al. reported the only existing data as to the impact of PCSK9 inhibitors on coronary calcification measured by Agatston calcium score (24). The study retrospectively evaluated 16 patients with PCSK9 inhibitors and 15 patients with statin monotherapy. The study showed that the annual CAC progression with the addition of the PCSK9 inhibitor to statin therapy (14.3%) is significantly lower than that with statin monotherapy (29.7%). The study is the first indicating CAC could be prevented by PCSK9 Inhibitors. However, the selection bias might present and the medical adherence cannot be assured in this study due to its retrospective non-randomized nature. Our study, on the other hand, overcame these limitations by conducting the calcium score analysis of a randomized controlled study. The results of our study also confirmed the potential role of PCSK9 inhibitor in inhibiting coronary calcification. However, the previous retrospective study showed greater extent of CAC progression than this study (24). Of note, compared with previous study, patients in our study had more advanced atherosclerosis and coronary calcification. Nearly 40% of patients in our study had baseline CAC score ≥ 400. In subgroups analysis with baseline CAC score ≥400 or <400, the effect of alirocumab on preventing CAC progression remained consistent, while the numerical rate of CAC progression was considerably lower for subjects with advanced CAC score.

Another interesting issue is the changes of plasma lipoproteins and CAC progression. The role of Lp(a) is well-established for the risk of coronary artery disease (25, 26), and several observational cohort studies demonstrated a positive association between Lp(a) levels and CAC (7, 9). Although statins have been documented with a beneficial effect on lipids, it has little impact on the Lp(a) levels. Contrary to the statins, PCSK9 inhibitors were reported to reduce plasma Lp(a) levels (26, 27). The results of current study also confirmed that the use of PCSK9 inhibitors was associated with significantly reduced Lp(a) levels, and the extent of CAC progression was correlated with the level of Lp(a) changes. Therefore, it seems reasonable to deduce that the effects of PCSK9 inhibitors on coronary calcification might be partially mediated by Lp(a) lowering effect. However, the exact mechanism of alirocumab on coronary calcification remains unknown. Whether is a direct effect of PCSK9 inhibition or a consequence mediated by Lp(a) reduction is still uncertain, and it warrants further randomized studies.

There are several potential limitations to this study. First, the progression of CAC could be influenced by some potential confounding factors, such as age, gender and traditional cardiovascular risk factors. However, due to limited sample size, stratified analyses with cardiovascular risk factors and biochemical parameters could not be carried out in this study. Subgroup analyses on the effects of statin dose adjustments and combined lipid lowering therapy cannot be performed as well. Second, the current study was a secondary analysis of a randomized study (15), in which CTA was not mandatary to all patients at baseline, and therefore it might introduce some potential bias into the study. Third, the relatively small number of patients in each group also limited the power of the study. Information concerning cardiovascular outcomes and its relation on CAC progression cannot be assessed in this study, because of the relatively short follow up period and the limited number of events. Further studies with lesion specific information and the clinical outcomes over time on larger numbers of subjects are urgently needed.

## Conclusions

The study showed administration of the PCSK9 inhibitors to statin-treated patients with coronary artery disease produced significantly lower rate of CAC progression, and the results might be partially related to the Lp(a) lowering effects with PCSK9 inhibitors.

## Data Availability Statement

The raw data supporting the conclusions of this article will be made available by the authors, without undue reservation.

## Ethics Statement

The study protocol was approved by Ethics Committee of Beijing Anzhen Hospital, Capital University. The patients/participants provided their written informed consent to participate in this study.

## Author Contributions

FG and YZ were responsible for the study design and management. DS were responsible for the data resources and integrity. ZW and XM conducted the OCT imaging analysis. YL helped with data management and statistical analysis. FG analyzed the data and drafted the manuscript. All authors read and approved the final manuscript.

## Funding

This study was funded by the Capital's Funds for Health Improvement and Research and the National Natural Science Foundation of China (82100349).

## Conflict of Interest

The authors declare that the research was conducted in the absence of any commercial or financial relationships that could be construed as a potential conflict of interest.

## Publisher's Note

All claims expressed in this article are solely those of the authors and do not necessarily represent those of their affiliated organizations, or those of the publisher, the editors and the reviewers. Any product that may be evaluated in this article, or claim that may be made by its manufacturer, is not guaranteed or endorsed by the publisher.

## Abbreviations

LDL: Low-density lipoprotein
PCSK9: proprotein convertase subtilisin kexin type 9
CAD: coronary artery disease
CAC: coronary artery calcification
CRP: C-reactive protein
Lp(a): lipoprotein(a)
ACEI/ARB: angiotensin-converting enzyme inhibitors/angiotensin receptor blockers
CTA: computed tomographic angiography.

## References

[1] Nakahara T Dweck MR Narula N Pisapia D Narula J Strauss HW. Coronary artery calcification: from mechanism to molecular imaging. JACC Cardiovasc Imaging. (2017) 10:582–93. 10.1016/j.jcmg.2017.03.005 28473100

[2] Uddin SMI Mirbolouk M Kianoush S Orimoloye OA Dardari Z Whelton SP. Role of coronary artery calcium for stratifying cardiovascular risk in adults with hypertension. Hypertension. (2019) 73:983–9. 10.1161/HYPERTENSIONAHA.118.12266 30879359 PMC6458064

[3] Puri R Nicholls SJ Shao M Kataoka Y Uno K Kapadia SR. Impact of statins on serial coronary calcification during atheroma progression and regression. J Am Coll Cardiol. (2015) 65:1273–82. 10.1016/j.jacc.2015.01.036 25835438

[4] Andelius L Mortensen MB Norgaard BL Abdulla J. Impact of statin therapy on coronary plaque burden and composition assessed by coronary computed tomographic angiography: a systematic review and meta-analysis. Eur Heart J Cardiovasc Imaging. (2018) 19:850–58. 10.1093/ehjci/jey012 29617981

[5] Bergmark BA O'Donoghue ML Murphy SA Kuder JF Ezhov MV Ceška R. An exploratory analysis of proprotein convertase subtilisin/kexin type 9 inhibition and aortic stenosis in the FOURIER trial. JAMA Cardiol. (2020) 5:709–13. 10.1001/jamacardio.2020.0728 32347887 PMC7301224

[6] Zhao X. Zhang HW Li S Zhang Y Xu RX Zhu CG. Association between plasma proprotein convertase subtisilin/kexin type 9 concentration and coronary artery calcification. Ann. Clin. Biochem. (2018) 55:158–64. 10.1177/0004563217695351 28166668

[7] Alonso R Mata P Muñiz O Fuentes-Jimenez F Díaz JL Zambón D. PCSK9 and lipoprotein(a) levels are two predictors of coronary artery calcification in asymptomatic patients with familial hypercholesterolemia. Atherosclerosis. (2016) 254:249–53. 10.1016/j.atherosclerosis.2016.08.038 27594539

[8] Perrot N Vincenza V Moschetta D Boekholdt SM Dina C Chen HY. Genetic and *in vitro* inhibition of PCSK9 and calcific aortic valve stenosis. J Am Coll Cardiol Basic Trans Science. (2020) 5:649–61. 10.1016/j.jacbts.2020.05.004 32760854 PMC7393433

[9] Greif M Arnoldt T von Ziegler F Ruemmler J Becker C Wakili R. Lipoprotein(a) is independently correlated with coronary artery calcification. Eur J Intern Med. (2013) 24:75–9. 10.1016/j.ejim.2012.08.014 23021791

[10] Schwartz GG Steg PG Szarek M Bhatt DL Bittner VA Diaz R. Alirocumab and cardiovascular outcomes after acute coronary syndrome. N Engl J Med. (2018) 379:2097–107. 10.1056/NEJMoa1801174 30403574

[11] Michos ED McEvoy JW Blumenthal RS. Lipid management for the prevention of atherosclerotic cardiovascular disease. N Engl J Med. (2019) 381:1557–67. 10.1056/NEJMra1806939 31618541

[12] Nicholls SJ Puri R Anderson T Ballantyne CM Cho L Kastelein JJ. Effect of evolocumab on progression of coronary disease in statin-treated patients: the GLAGOV randomized clinical trial. JAMA. (2016) 316:2373–84. 10.1001/jama.2016.16951 27846344

[13] Ako J Hibi K Tsujita K Hiro T Morino Y Kozuma K. Effect of alirocumab on coronary atheroma volume in Japanese patients with acute coronary syndrome - the ODYSSEY J-IVUS trial. Circ J. (2019) 83:2025–33. 10.1253/circj.CJ-19-0412 31434809

[14] Shekar C Budoff M. Calcification of the heart: mechanisms and therapeutic avenues. Expert Rev Cardiovasc Ther. (2018) 16:527–36. 10.1080/14779072.2018.1484282 29860888 PMC6309454

[15] Gao F Wang ZJ Ma XT Shen H Yang LX Zhou YJ. Effect of alirocumab on coronary plaque in patients with coronary artery disease assessed by optical coherence tomography. Lipids Health Dis. (2021) 20:106. 10.1186/s12944-021-01528-3 34511134 PMC8436513

[16] Friedewald WT Levy RI Fredrickson DS. Estimation of the concentration of low-density lipoprotein cholesterol in plasma, without use of the preparative ultracentrifuge. Clin Chem. (1972) 18:499–502. 10.1093/clinchem/18.6.499 4337382

[17] Hecht HS Cronin P Blaha MJ Budoff MJ Kazerooni EA Narula J. 2016 SCCT/STR guidelines for coronary artery calcium scoring of noncontrast noncardiac chest CT scans: a report of the Society of Cardiovascular Computed Tomography and Society of Thoracic Radiology. J Cardiovasc Comput Tomogr. (2017) 11:74–84. 10.1016/j.jcct.2016.11.003 27916431

[18] Agatston AS Janowitz WR Hildner FJ Zusmer NR Viamonte M Jr Detrano R. Quantification of coronary artery calcium using ultrafast computed tomography. J Am Coll Cardiol. (1990) 15:827–32. 10.1016/0735-1097(90)90282-T 2407762

[19] Akers EJ Nicholls SJ Di Bartolo BA. Plaque calcification: do lipoproteins have a role? Arterioscler Thromb Vasc Biol. (2019) 39:1902–10. 10.1161/ATVBAHA.119.311574 31462089

[20] Jinnouchi H Sato Y Sakamoto A Cornelissen A Mori M Kawakami R. Calcium deposition within coronary atherosclerotic lesion: Implications for plaque stability. Atherosclerosis. (2020) 306:85–95. 10.1016/j.atherosclerosis.2020.05.017 32654790

[21] Lupo MG Bressan A Donato M Canzano P Camera M Poggio P. PCSK9 promotes arterial medial calcification. Atherosclerosis. (2022) 346:86–97. 10.1016/j.atherosclerosis.2022.01.015 35135698

[22] Yano H Horinaka S Ishimitsu T. Effect of evolocumab therapy on coronary fibrous cap thickness assessed by optical coherence tomography in patients with acute coronary syndrome. J Cardiol. (2020) 75:289–95. 10.1016/j.jjcc.2019.08.002 31495548

[23] Adamson PD Newby DE. Non-invasive imaging of the coronary arteries. Eur Heart J. (2019) 40:2444–54. 10.1093/eurheartj/ehy670 30388261 PMC6669405

[24] Ikegami Y Inoue I Inoue K Shinoda Y Iida S Goto S. The annual rate of coronary artery calcification with combination therapy with a PCSK9 inhibitor and a statin is lower than that with statin monotherapy. NPJ Aging Mech Dis. (2018) 4:7. 10.1038/s41514-018-0026-2 29951223 PMC6015059

[25] Burgess S Ference BA Staley JR Freitag DF Mason AM Nielsen SF. Association of LPA variants with risk of coronary disease and the implications for lipoprotein(a)-lowering therapies: a mendelian randomization analysis. JAMA Cardiol. (2018) 3:619–27. 10.1001/jamacardio.2018.1470 29926099 PMC6481553

[26] Ruscica M Sirtori CR Corsini A Watts GF Sahebkar A. Lipoprotein(a): knowns, unknowns and uncertainties. Pharmacol Res. (2021) 173:105812. 10.1016/j.phrs.2021.105812 34450317

[27] O'Donoghue ML Fazio S Giugliano RP Stroes ESG Kanevsky E Gouni-Berthold I. Lipoprotein(a), PCSK9 inhibition, and cardiovascular risk. Circulation. (2019) 139:1483–92. 10.1161/CIRCULATIONAHA.118.037184 30586750

